# “It Was Changing [My] Embedded Inner Culture”: Culturally Informed Training in STEMM

**DOI:** 10.3390/educsci16030427

**Published:** 2026-03-01

**Authors:** Judith C. P. Lin, Carrie L. Saetermoe, Sophia E. Lucas, Armando Gonzalez, David Boyns, Yolanda Vasquez-Salgado, Shu-Sha Angie Guan

**Affiliations:** 1 Educational Modules to Broaden Academic Research Cultures, Health Equity Research and Education Center, California State University, Northridge, Northridge, CA 91330, USA; 2 Department of Psychology, California State University, Northridge, Northridge, CA 91330, USA; 3 Department of Sociology, California State University, Northridge, Northridge, CA 91330, USA; 4 Department of Child and Adolescent Development, California State University, Northridge, Northridge, CA 91330, USA

**Keywords:** STEMM education, community college students, cultural mismatch, community cultural wealth, sense of belonging, science identity, self-efficacy, mentorship, STEMM careers, hidden curriculum

## Abstract

While scholars have written about programs that support community college (CC) students in STEMM and their transition to four-year institutions, less attention has been paid to culturally informed approaches addressing cultural mismatch and leveraging community cultural wealth (CCW). This paper presents results from qualitative research conducted through five focus groups with 43 CC students who attended a summer program over three summers. Grounded in strategies that address cultural mismatch by drawing on CCW and ancestral strengths to foster STEMM success, the summer program provides CC students with professional tools and skills for their educational trajectories and supports them as they pursue a STEMM pathway. The findings revealed that learning about multiple pathways through faculty members’ academic journeys, bonding with peers, and gaining the language to describe home–school mismatch experiences allowed students to reduce intimidation and self-blame, increase self-efficacy, develop a stronger science identity invested in social justice, and demonstrate greater willingness to engage with institutional actors. In sum, humanizing STEMM not only portrays a holistic picture of the field but also demystifies the notion of STEMM as a “cold” career, enabling students to more readily see themselves in role models and envision themselves on a viable career path.

## 1. Introduction

### 1.1. Background and Context

#### 1.1.1. The Value of Untapped Human Capital

Community colleges (CCs) serve a heterogeneous constituency and represent a gold-mine of untapped potential. Their diversity holds immense promise for strengthening the workforce in Science, Technology, Engineering, Mathematics, and Medicine (STEMM), enhancing capacity for innovation and global competitiveness. According to the Community College Research Center, CCs enrolled 8.6 million students in 2022–2023, accounting for 40% of all undergraduate students enrolled in the U.S. that year (Community College Research Center, n.d.). Nationally, roughly 50% of all Hispanic undergraduates were enrolled at CCs, compared with 40% of Asian, 39% of Black, and 38% of white undergraduates. Additionally, the average age of CC students is 27 (median = 23); about 32% students enrolled at CCs are first-generation college students, 4–5% are veterans, and about 23% report having one or more disabilities (American Association of Community Colleges, n.d., 2025).

CCs fulfill an array of educational, vocational, and civic functions. Among their many roles, CCs offer opportunities to explore career options and to meet pre-career objectives. However, as funding agencies more readily see a payoff at the graduate and postdoctoral levels (e.g., increasing diversity among physicians or professors), CCs have long been overlooked as sources of STEMM researchers and practitioners (Snyder & Cudney, 2017), resulting in limited research and training infrastructure and poor funding for CCs (Cejda, 2019; NASEM, 2016). This approach neglects a vast, untapped pool of talents at the CC level—individuals who have the potential to significantly strengthen the US STEMM pipeline. In addition to limited resources, demanding teaching responsibilities have been recognized as a major obstacle for faculty who wish to offer research opportunities (Hewlett, 2019). Supporting collaborations between CCs and four-year institutions is therefore a critical contribution to both science and healthcare (Judge et al., 2022; NASEM, 2017; Olson & Riordan, 2012), as these partnerships help CC students—particularly those from historically underrepresented backgrounds—persist and succeed in their STEMM journey.

Diversifying the biomedical research and professional workforces is essential not only for improving healthcare outcomes but also for unlocking untapped innovative potential. Studies show that minority and women physicians are significantly more likely than their white peers to practice in underserved communities and to care for minority, low-income, and Medicaid patients—helping to expand access to care where it is needed most (Cantor et al., 1996; Cohen et al., 2002; Institute of Medicine, 2004). Further, patients from minority backgrounds tend to prefer, and report greater satisfaction with, physicians who share their racial or ethnic background, highlighting the importance of workforce diversity (Cohen et al., 2002; LaVeist & Nuru-Jeter, 2002; Institute of Medicine, 2004). Another study has found that if women, minorities, and children from low-income families engaged in invention at the same rate as white men from high-income families, the number of inventors in the US would quadruple, a transformation that could significantly boost economic growth (Bell et al., 2019).

#### 1.1.2. Continuing Challenges of the Leaky Pipeline

At the level of CCs, one of the most effective ways to promote health equity is by ensuring equal access and support for traditionally underrepresented students in STEMM research and practice. Despite significant investments aimed at diversifying the STEMM workforce, women and traditionally underrepresented racial and ethnic groups continue to lag their peers in STEMM fields. As of 2021, only 18% of women were employed in STEM occupations, roughly three-fifths the rate of men (30%); and Black or African American (8%) and Hispanic (15%) workers were underrepresented in STEM occupations compared to their share of the overall workforce (11% and 17%, respectively; National Science Board, National Science Foundation, 2024). These disparities can be partially attributed to patterns in STEM degree attainment. While women are overrepresented in certain STEM fields, such as health-related and life sciences, they remain underrepresented in mathematics, physical sciences, computing, and engineering (Fry et al., 2021). Racial and ethnic disparities also persist in STEM education. For example, in 2018, Hispanic adults earned just 9% of STEM master’s degrees and 6% of STEM research doctorates—lower than their shares of total master’s (11%) and research doctorate degrees (8%) across all fields. Similarly, Black students earned no more than 9% of STEM degrees at the bachelor’s, master’s, and doctoral levels, a figure that has seen little change since 2010 and remains lower than their share of total degrees earned across all fields at each of these educational levels (Fry et al., 2021). Furthermore, among individuals with at least a bachelor’s degree in a science and engineering (S&E) field, many do not work in STEM-related occupations. Specifically, 60% of women and 58% of Black or African American degree holders with an S&E background were employed outside of S&E or S&E-related roles, contributing to the leaky STEMM pipeline (National Science Board, National Science Foundation, 2024).

This leaky pipeline is also evident at the CC level, where STEM attrition is significantly higher among associate’s degree students (69%) compared to bachelor’s degree students (48%). Moreover, proportionally more Black and Hispanic students leave STEM fields—either by dropping out of college or switching to a non-STEM major—compared to their white and Asian counterparts, making them a smaller share of CC students who transfer and complete a STEM bachelor’s degree (Chen & Soldner, 2013; Meza, 2019; NASEM, 2016).

These general patterns suggest that while traditionally underrepresented students often aspire to attend a four-year college, they face significant challenges in connecting to four-year institutions and pursuing STEMM fields of study. These challenges are especially pronounced for first-generation college students whose parents have had limited exposure to higher education (Chen, 2005). Additionally, CC students in STEMM tend to be older, are more likely to work while enrolled, and often work more hours when working compared to their peers at four-year institutions (NASEM, 2016). Multiple social roles and limited resources at the institutional and personal level explain why CCs have the lowest STEMM retention rates (American Association of Community Colleges, 2025; Snyder & Cudney, 2017). Additional barriers such as transfer stigma, lack of institutional support, tuition cost, and limited access to research opportunities further impact their ability to succeed and persist in STEMM disciplines (NASEM, 2016).

#### 1.1.3. Peer Navigators as a Key Support Mechanism

Given the complexity of their objectives and student populations, tailored approaches that address the needs and cultures of the CC population are needed to successfully retain students in STEMM fields, both before and after transfer (Snyder & Cudney, 2017). One promising approach is the implementation of peer navigator programs, which not only help alleviate faculty workload but also foster a stronger sense of belonging, identity, and purpose among students pursuing STEMM pathways. Peer navigators in STEMM fields—particularly those from similar backgrounds—can effectively engage a student body that is often composed of first-generation college students. These peer navigators can help guide their peers through the “hidden curriculum” of higher education, validate their sense of belongingness and science identities in STEMM fields, and serve as a bridge to support their academic and social success (Beals et al., 2021; Bowling et al., 2015; Rockinson-Szapkiw et al., 2021; Zaniewski & Reinholz, 2016). Peer navigators also benefit from supporting their peers. Reported gains include shifts in their perceptions on teaching and learning; enhanced leadership, communication, and presentation skills; strengthened identity development; and improved academic outcomes (Bowling et al., 2015; Spaulding et al., 2020).

While scholars have written about programs that support community college (CC) students in STEMM and their transition to four-year institutions, less attention has been paid to culturally informed approaches addressing cultural mismatch and leveraging community cultural wealth. This is the gap that Educational Modules to Broaden Academic Research Cultures (EMBARC) and this paper seek to address. EMBARC is a peer-led program for pre-transfer CC students in the STEMM fields (Guan et al., 2025). The program equips CC students with the skills and resources necessary for academic and research success through professional development workshops, transfer preparation, and community building. Through a series of workshops and mentorship grounded in community cultural wealth frameworks that embrace students’ cultural strengths, EMBARC supports CC students as they navigate STEMM majors and their transition to four-year institutions. These frameworks are intended to increase STEMM transfer readiness and foster harmonization between home and academic cultures, enabling students to make meaningful connections between their science courses and family activities.

### 1.2. Theoretical Framework

#### 1.2.1. Community Cultural Wealth

In her widely cited paper, Yosso (2005) elevates the cultural knowledge, skills, and networks possessed by socially marginalized groups—assets that are often unrecognized or overlooked. She outlines six forms of capital cultivated through community cultural wealth (CCW)—aspirational, navigational, social, linguistic, familial, and resistant capital—affirming the rich knowledge and insight that students carry from their home communities into the educational space.

At a foundational level, Yosso’s contribution is philosophical and epistemological: she challenges dominant, deficit-based frameworks by proposing a new way of knowing, understanding, and making meaning—one that centers the lived experiences and cultural assets of communities of color. Building on traditions of “outsider” knowledges (Collins, 1986), *mestiza* knowledges (Anzaldúa, 1987), and transgressive knowledges (Hooks, 1994), Yosso expands the epistemic boundaries of educational discourse and offers a critical framework for valuing non-dominant forms of cultural capital.

CCW thus serves as a valuable framework for understanding how students draw upon their cultural assets to navigate their educational journeys. From the pre-transfer stage where students build resources and networks to manage the complexities of the transfer process, to the post-transfer stage, where they confront challenges such as transfer shock, increased academic rigor, and limited institutional support, these forms of capital help them persist in pursuit of their STEMM goals.

#### 1.2.2. Cultural Mismatch, Ancestral Strength, and Cultural Identity

Cultural Mismatch Theory, made popular by the seminal work of Stephens et al. (2012), argues that the norms of independence embedded in the U.S. higher education system (e.g., being individually motivated) create a cultural mismatch for first-generation college students, many of whom come from interdependence cultural backgrounds that emphasize collaboration. The mismatch, as demonstrated through a series of experiments, can negatively impact first-generation students’ academic performance, sense of belonging, and overall well-being.

Building on the theory, Vasquez-Salgado et al. conducted multiple studies using qualitative, survey, and experimental methods (e.g., Vasquez-Salgado et al., 2021, 2022) and identified various forms of cultural mismatch experienced by first-generation college students, for example, conflicts between interdependent family obligations and independent academic obligations, and between their interdependent ideologies and practices and the ideologies and practices of their more independent peers (termed peer-peer cultural mismatch). The authors found that frequent experience with these mismatches negatively affected students’ mental and physical health, and poorer health was associated with academic problems and lower grades. More recently, they discovered that mismatch is associated with faster biological aging (Vasquez-Salgado et al., 2025), providing further credence on the need to devise culturally informed interventions that can work to harmonize home and academic cultures.

One of the few antidotes to cultural mismatch in education spaces is the adoption of culturally responsive practices, which argue that embracing one’s cultural identity does not hinder but enhances one’s academic and professional potential (Hall et al., 2023; Hutchison & McAlister-Shields, 2020; Lin et al., 2025; Macapugay & Nakamura, 2024; Warren et al., 2020). While underexplored in the literature, we propose that ancestral strength represents an emerging framework that can support students’ motivation, resilience, and persistence in STEMM pathways. Rooted in familial capital, a subcomponent of CCW theory, ancestral strength emphasizes reflection on one’s family ancestors (living or deceased) and the intergenerational resilience and spiritual grounding such reflection fosters. By contextualizing the strength of their ancestors as trailblazers through their lived experiences, this concept helps students recognize that they, too, can become trailblazers by pursuing STEMM education and careers, especially as many are the first in their families to attend college. Although cultural mismatch has been explored from various perspectives in previous research, studies have not examined the viewpoints of training recipients through these theoretical frameworks, particularly capturing students’ voices via focus groups. This study therefore contributes to the literature by directly highlighting students’ experiences and perceptions.

#### 1.2.3. Liberation Psychology as a Path Forward

Liberation Psychology (Martín-Baró et al., 1994; Vargas & Saetermoe, 2024) provides a framework in which transformation takes place in the process of looking backward to gain knowledge that deconstructs prior beliefs that are damaging (e.g., racism, internalized racism) and to reconstruct a new way of being, acted out through praxis. As analyses progressed in this study, a story emerged from students’ expressed experiences that fit a process of liberatory change: becoming aware of new realities (transfer, STEMM, role models), deconstructing old realities that were vague or discouraging, in part by acknowledging ancestral strengths, and then reconstructing a new pathway and acting on it (praxis). Having few role models and limited information, before EMBARC, students experienced a “community college mindset” that left them “stuck” in a vague fear of the future, self-doubt, and susceptible to pressures like cultural mismatch. Students were able to become aware of their status through exposure to role models and career pathways that allowed them to reconstruct a future that has a clear pathway, steps to get to a specific goal, and intention and action (praxis) to achieve their goal. Understanding the pathway to clarity and purpose requires the acknowledgement of one’s ancestral strengths and CCW and the specific means to employ these concepts to achieve educational and career goals.

It should be noted that while CCW, cultural mismatch, and ancestral strength functioned as program constructs that helped students articulate their lived experiences, Liberation Psychology served as the interpretive lens for this study, illuminating how students engaged with their inner culture before, during, and after the program.

### 1.3. The EMBARC Model

The EMBARC workshops are grounded in strategies that seek to address cultural mismatch by inviting students to recognize it, providing them with strategies for harmonization, and bringing their CCW (aspirational, linguistic, familial, social, navigational, resistant) to light, as well as delving deeper into their ancestral strengths to foster student STEMM success. In the first three years of our five-year grant program, EMBARC comprised three primary activities conducted throughout the year: (a) a cohorted summer intensive, face-to-face (SI-FTF) on-campus workshop series that develops a full range of skills in a resource-rich environment, (b) training of qualified students from the SI-FTF to become peer navigators, and (c) a series of in-person and online workshops delivered by peer navigators during the academic year.

The key components of SI-FTF are to provide CC students interested in biobehavioral careers with (1) formal modules introducing tools in harmonizing and forging a meaningful connection between their home and school cultures, (2) content on biobehavioral research careers, (3) professional development skills workshops, such as communicating with faculty, (4) training in research and technical skills, (5) didactic content on ethics and responsible conduct, (6) transfer support and intention building, and (7) mentoring in other skills that can support students in their health and academic adjustment to the university as well as in academic and research programs they pursue in the future. Providing CC students with these tools and skills early in their education trajectories can reduce experiences with cultural mismatch and increase science self-efficacy (Guan & Vasquez-Salgado, 2023). Students participating in the SI-FTF training completed an Individualized Development Plan (IDP) to set personal goals and track progress, as early goal-setting has been shown to promote productivity and performance (Davis, 2005; Hobin et al., 2014). They also received financial support in the form of participation stipends, on-campus housing, and per diems. Specific information about the modules and module development can be reviewed in Guan et al. (2025).

The SI-FTF training was followed by the EMBARC Training-the-Trainers (TTT) program. Selected trainees became trainers, or peer navigators, who then brought the one-hour modules back to their respective campuses across the academic year. Peer navigation is intentionally integrated into the EMBARC TTT model to empower students and promote an academic teaching-scholar approach, which can, in turn, strengthen research culture at their respective CC campuses (Garde-Hansen & Calvert, 2007). During the academic year, EMBARC peer navigators deliver workshops that provide CC students with a meaningful introduction to STEMM fields and to support them as they prepare for and pursue a STEMM pathway. Additionally, peer navigators participated in continued leadership and professional development across the year resulting in conference presentations across cohorts.

### 1.4. Study Purpose and Research Questions

In this qualitative study, we examined the cohorted SI-FTF on-campus workshop series by analyzing outcomes from focus groups conducted during the summers of 2023, 2024, and 2025. In particular, we examined how EMBARC supports student development through a program promoting STEMM exploration in CC students. Additionally, we explored how the training fosters students’ awareness of systemic structures through the lens of cultural mismatch, while also acknowledging and leveraging cultural strengths (e.g., ancestral strengths, CCW) embedded in students’ daily lives. The findings revealed that learning about multiple pathways through faculty members’ academic journeys, bonding with peers, and gaining the language to describe home–school mismatch experiences allowed students to reduce intimidation and self-blame, increase self-efficacy, develop a stronger science identity invested in social justice, and demonstrate greater willingness to engage with institutional actors. In doing so, this paper contributes new insights to the literature by addressing gaps in our understanding of cultural mismatch and the role of cultural identities in STEMM pathways, particularly for CC students. This study was guided by a research question that asks, *How does EMBARC impact students’ desire to pursue a STEMM career, their sense of belonging and ability to complete STEMM preparation, and their identity as a scientist or practitioner?*

## 2. Materials and Methods

### 2.1. Study Participants

Students who participated in summer training program in 2023 (*n* = 8), 2024 (*n* = 9), and 2025 (*n* = 26) took part in the study. The number of student participants increased as the program grew and expanded. During the first two years, students attended the summer training knowing they would become peer navigators for the following academic year. However, students in the third year did not share this mindset, as navigators were selected only after the program concluded. Because the cohort size had a more direct impact on students’ experiences than the participants’ role as peer navigators, the nature of student participation (whether or not they entered the program as peer navigators) was not the focus of our analyses.

All students who started the summer program completed it. As part of the program, students participated in focus groups at the conclusion of their EMBARC summer experience. There was one focus group in each of the first two years, and three in the third year. A total of 43 summer students participated in five focus groups over three years. The evaluation was conducted by our external evaluator. All research instruments and related protocols were approved by the Institutional Review Board (IRB) at California State University, Northridge. Demographic characteristics of the participants are displayed in Table 1.

### 2.2. Focus Group

To collect the data, semi-structured focus group interviews with 8–10 participants were conducted, using seven main questions to explore students’ experiences of the EMBARC program. The discussion centered on the activities and modules, personal and academic growth, cultural relevance, group experiences, and the benefits of living on campus. Each focus group lasted about 90 min, was audio recorded, transcribed, and then analyzed for common themes. Because focus groups can heighten social desirability bias, facilitators emphasized that there were no “right” answers, explicitly invited both positive and negative feedback, and used neutral prompts during the interviews. Participants were also reminded not to share identifying remarks outside the group and were offered the option to provide additional comments privately after the session. Audio recordings of the focus group interviews were made after obtaining the participants’ prior permission, ensuring their confidentiality. In cases where students declined to be audio recorded (*n* = 1), the researchers stopped the recorder whenever the student spoke.

### 2.3. Data Analysis

Before beginning thematic analysis, all coders met to discuss constructivist grounded theory methodology and methods (Charmaz, 2014). Thematic analysis of data (open coding, Charmaz, 2014; Strauss & Corbin, 1998) were conducted by the first four authors of this paper who employed constant comparison between incidents, times, and type of respondent to: (1) review each focus group transcript, (2) write memos for each transcript, and (3) identify initial themes based on a starting rubric from the interview schedule (programmatic benefits, changes, cultural content, transfer issues, barriers, and people), looking for change before and after the program, and factors that contribute to interest and commitment to a STEMM career and beliefs that one can complete the education required for a STEMM career. Secondary memos were generated (between 2 and 8 per initial theme), intended to be synthetic, connective, and linked to outcomes. In the final stage of independent coding, each of four coders identified two to eight themes, wrote formal definitions, found three examples from the transcripts of the theme, and related the theme to changes and STEMM interest and educational preparation.

Independent coding was followed by a group discussion to negotiate inter-coder triangulation (Richardson & St. Pierre, 2005) in content and relevance of themes and to identify a higher-order framework across areas that allowed for thick description, a deeper analysis of underlying processes. A Liberation Psychology framework (Martín-Baró et al., 1994; Vargas & Saetermoe, 2024) was proposed to the group by the second author, and coders read an article about its application in teaching to examine themes across coders (Saetermoe et al., 2025). After this discussion, coder writing assignments for thematic areas around programmatic effectiveness and student transformation through Liberation Psychology phases were made by the first author. For example, in Table 2, where final themes can be found, two secondary themes emerged under the rubric of Guidance, Mentorship, and Navigation: students deconstructed their own experiences of alienation with education by understanding the role of structural factors in their experiences (racism, institutional size) and reconstructed a new pathway alongside of their faculty or other mentor that takes into account their community cultural wealth and passions.

### 2.4. Researchers’ Positionality

The five authors who conducted the data analysis and reported results came from diverse backgrounds, and their roles and involvement in the program varied.

JCPL, an Asian researcher with a background in education, has worked primarily in the research wing of EMBARC, interacting with students only intermittently during the third-year summer program. This distance allows for a degree of objectivity, while her commitment to deep listening to the text ensures that student perspectives remain central to her interpretation. She did not attend a community college, and, aware that her personal experiences could shape her understanding, she consciously sets aside assumptions and lets the students’ voices guide her analysis. This reflexive approach enhances the trustworthiness and validity of her findings.

CS, a white professor of developmental psychology and qualitative methods has been principal investigator on several educational grants including the EMBARC grant. Her direct involvement with students in EMBARC has been social in nature. Personal experiences in community college, along with teaching and research experiences were, as much as possible, partitioned from the current data by listening naively for voices emanating from across age, racial/ethnic, sex, and other identities; transfer and scholarly contributions were included in group analysis.

SL, a Latina graduate student in the Sociology department, is a first-generation college student and attended a community college in her education path. She works primarily in education, social research, program evaluation, and public service. This background has allowed her to resonate with student backgrounds while maintaining an evaluator’s distance from participants to provide objective analysis. Similarities and differences in personal and participant experiences are reflected throughout the research process, allowing for a degree of internal knowledge that benefits program improvement and an external acknowledgment of the importance of highlighting all participant voices in the findings.

AG, a Mexican American male completing a master’s degree in sociology, brings both lived experience and sociological training to this research. His background sensitizes him to structural inequality, institutional culture, and educational stratification, particularly for students from historically marginalized communities. His own journey in higher education informs how he interprets participants’ reflections on belonging, cultural mismatch, and identity. His professional role as an evaluation researcher shapes his analytic lens, leading him to attend to program structures and outcomes. Throughout the process, he engaged in collaborative coding and reflexive dialogue to ensure that findings remained grounded in participants’ narratives rather than his own expectations.

DB, a white professor of Sociology, served as the lead external evaluator for study and was responsible for study data collection and consenting of participants. As an evaluator operating outside the program’s day-to-day operations, he brought relative independence to the study while also occupying a position of perceived authority. His extensive experience advising and teaching underrepresented students, as well as his position external to the program, allowed him to empathize with students, as well as reduce social desirability and power dynamics. He emphasized voluntary participation, clarified that responses would not affect students’ program standing or access to services, and used neutral prompts that explicitly invited both positive and negative perspectives.

## 3. Results

Qualitative data analysis revealed seven key themes (Table 2) across five focus groups, as follows: (1) guidance, mentorship, and navigation, (2) acquisition of knowledge, hard skills, and the hidden curriculum, (3) peer bonding and social support, (4) faculty-student interactions and relationships, (5) navigating cultural mismatch, (6) benefits of on-campus living, and (7) validation and commitment to STEMM. Within each of the major themes, several sub-themes were identified. Sub-themes will be discussed within each theme, presented through the lens of Liberation Psychology, addressing the social positioning of students, the processes, and the impacts.

### 3.1. Guidance, Mentorship, and Navigation

#### 3.1.1. Dissonance Without Structural Frameworks

Before entering EMBARC, many students described feeling uncertain, hesitant, and internally conflicted about their educational paths. Several participants entered the program without a clear understanding of how to navigate higher education systems, including research opportunities, transfer pathways, or professional engagement. Dorothy, an Asian American participant reflected, “Most of the times I do feel lost or panic about whether I’m doing the right thing to help me stay on track.” This lack of guidance was often internalized as self-doubt rather than recognized as a structural gap within inequitable educational systems. First-generation and transfer-intending cohort members, in particular, expressed feeling intimidated by faculty and professionals, unsure of how to communicate with individuals in positions of authority, and uncertain whether they belong in academic or professional spaces. Such uncertainty reflects not individual deficiency but limited access to affirming, navigational relationships with institutional ecosystems. Furthermore, students described entering the program without language to explain the tension they experienced between family expectations and educational aspirations (see more discussion on Section 3.5 below). Without frameworks to name cultural mismatch, hidden curriculum, or structural barriers, some participants internalized guilt or felt selfish for prioritizing school. These experiences left students feeling isolated, cautious, and unsure whether they were “on the right path,” particularly those navigating nontraditional or nonlinear trajectories. Reported feelings reflected dissonance and discomfort, but lacked the conceptual tools to situate them within broader systems of inequality.

#### 3.1.2. Guidance as Meaning Making: From Passive Receipt to Active Co-Construction

During EMBARC, students encountered guidance that extended beyond procedural instruction and into internal meaning-making. Rather than simply offering information, the program provided relational, reflective mentorship that actively demystified higher education systems while simultaneously addressing emotional and identity-based barriers (see discussion on Section 3.4). Beyond individual mentorship, the program functioned as a guiding ecosystem. Students were introduced to multiple pathways simultaneously—research, transfer, graduate school, and careers—without being forced into a singular or linear trajectory. Students emphasized that this exposure reframed guidance as developmental rather than prescriptive, and that a wide range of STEMM fields helped them reimagine uncertainty as exploration. For example, Christopher, a Latino participant, stated:

“I learned more about what I can potentially do in the future. But for someone who comes to college undecided, they get introduced to all these fields, including STEMM fields, which is really interesting. … It’s a guiding program. It guides people.”

Faculty and mentors also actively demystified access points into research and professional spaces. Robert, a Latino participant, described this process as a “gateway,” stating,

“I would say gateway. I always want to get into research, but I didn’t know where to start, who to reach out to, or how to. And a lot of the faculty and people outside … were really open to giving us a gateway or an access point into research positions or volunteering.”

Following the program, students appeared to have reconstructed their understanding of guidance as something they could actively seek, co-create, and sustain. Rather than viewing uncertainty as personal failure, cohort members began to recognize it as a navigational challenge that could be addressed through relationships, mentorship, and structural knowledge. This shift was associated with indications of increased self-efficacy, reduced intimidation, and greater willingness to engage with institutional actors.

### 3.2. Acquisition of Knowledge, Hard Skills, and the Hidden Curriculum

#### 3.2.1. Anxiety and Self-Doubt Stemming from Limited Access

Prior to participating in EMBARC, many students entered with limited access to the hidden curriculum of higher education. While some demonstrated strong academic motivation, they lacked exposure to professional norms, research practices, and transferable skills typically expected at four-year institutions. Community college students, in particular, described feeling unprepared for activities such as professional communication, public speaking, networking, and research design. This lack of access often manifests as anxiety around competence and fear of judgment. Several participants expressed uncertainty about their skill sets and doubted their ability to perform in professional or academic settings. Without structured opportunities to practice these skills, students entered the program unsure of how to translate their interests into concrete action.

#### 3.2.2. Expanding Academic Capacity Through Exposure to Diverse Tools and Skills

During the program, EMBARC provided students with tangible, applied skill-building experiences that directly addressed these gaps. Participants engaged in activities such as creating elevator pitches, developing research questions, conducting data analysis, and building professional materials (CVs, LinkedIn profiles). These experiences were framed not as evaluations but as opportunities for growth within a supportive, nurturing environment. Cohort members such as Laura, a Latina participant, detailed how exposure to a variety of tools and skills expanded her understanding of academic work:

“I learned how to create the correct research question…the whole research process. I also really am enjoying the data analysis…. Those are very helpful tools for us. I think as students who are trying to get into different fields, it’s very important for us to now be able to also have our digital resume on LinkedIn.”

Skill-building activities foster confidence through productive challenges. Presentations, project development, and reflective exercises provided opportunities for students to test and expand perceived limits in supportive contexts. Emerson, a Latina student, reflected on how being placed under pressure revealed previously unrecognized resilience,

“I think being put under pressure showed me how, from a personal point of view, I can be resilient…. I realized that I actually could do that, so I feel like it helped me gain more confidence in myself.”

After the program, students reported a noticeable increase in confidence rooted in both skill mastery and self-recognition. By acquiring practical tools and understanding institutional norms, students felt better equipped to navigate inequitable systems and advocate for themselves. The integration of hard skills, hidden curriculum knowledge, and reflective frameworks supported students in reconstructing their identities as capable, resilient, and deserving of access within higher education.

### 3.3. Peer Bonding as a Foundation for Belonging

Entering a new program, meeting new people, and staying away from home with strangers for two weeks was anxiety-provoking for students, some of whom reported feeling scared upon entering the program. This anxiety was quickly alleviated through meaningful connections with peers in the safe spaces facilitated by the program.

#### 3.3.1. Vulnerability Fosters Emotionally Powerful Connections

Discussions of ancestral strengths and community cultural wealth accelerated peer bonding, as students embraced fear, took emotional risks, and shared their personal stories. In being seen and heard, the storyteller experiences a form of relational safety that not only affirms their vulnerability but also invites deeper connection. This experience of being held and understood, in turn, cultivates a willingness to extend the same empathy, care, and openness toward others, strengthening the collective bond within the group. This sense of vulnerability facilitated further openness and sharing, creating emotionally powerful connections among group members. Andrew, a Latino participant, identified the Ancestral Strengths workshop as one of his highlights, commenting that the cohort was going to bond through discussing their own stories “*whether you like it or not*,” implying that the cultural discussions themselves intrinsically have the ability to create connections.

#### 3.3.2. From Bonding to Belonging Through Trust and Safety

During the program, students found solace in others’ stories, knowing that they are not alone in their journeys. At the same time, students bonded by embracing differences that foster mutual growth. The trusting relationships provided students with a sense of safety, allowing them to remain open while being challenged by the differences observed within the group, whether related to family or academic backgrounds. The mutual trust and safety translated into a sense of belonging, even for those who were accustomed to fending for themselves. Kevin, a Latino student, acknowledged, “*But then when I left and you guys were worried about where I was, that made me feel like, oh, you know, I belong here*.” A strong sense of belonging encouraged collective progress across program components, which further reinforced students’ belonging. In the first two, smaller cohorts, this evolved into a family-like cohesion, as captured by Darby, a female Black/African American participant:

“I feel like my best experience is getting to know everyone here, getting to know the different personalities, getting positive feedback, getting the negative feedback, the constructive criticism and building a family from the ground up basically and learning different modules that we have not been aware of before. Just realizing some social issues and being able to present them with our EMBARC family, our first EMBARC family, that was the best part for me.”

Individual EMBARC students may have started with personal goals in mind, but these developed into a sense of community effort and group success.

#### 3.3.3. From Belonging to Embracing Challenges in Team Science

In an anchored environment, students recognized and affirmed the benefits of team science, appreciating the value of diverse groups working together. When Darby (Female, Black/African American) stated, “I like that they challenged me,” multiple students echoed her sentiment. Thomas, a Latino participant who usually works alone and does not typically collaborate with others, acknowledged that, “*All of the group activities* … *changed that for me. [I]t made me want to do it with other people because it felt that much better accomplishing or just finishing in general*.” Thomas also pointed out that the program was “*changing embedded inner culture*” for him, reflecting a shift in students’ internal functioning. Through thoughtfully structured group activities, students appeared to have internalized the principles of team science.

### 3.4. Mindset Shifts: Preparing for the Future and Faculty-Student Relationships

#### 3.4.1. Unlocking the Potential of Faculty-Student Relationships: Openings for the Future

A substantial transition took place in students’ sense of faculty characteristics, accessibility, and supportiveness before and after participation in EMBARC training. As a result of the shift in faculty perception, students expressed a new openness to their possibilities with STEMM as well as a renewed confidence and conviction that, with the support of a community of faculty, professionals, and peers, they, too, could reach the goals achieved by their role models.

Before EMBARC, faculty were often seen as unapproachable, uninterested in student needs, uninspired or arrogant in their research, or privileged in their linear progression to becoming professors. By exposing students to faculty members and STEMM-related professionals who were passionate about teaching, medicine, and research, their sense of faculty members was deconstructed and replaced with relationships characterized by casual approachability, humanized stories of nonlinear pathways, and new perceptions of possible student-faculty relationships that involve sharing opportunities, resources, networks, and discipline-based skills.

The primary PIs, the sixth and seventh authors of the paper, were often mentioned as inspirational, thoughtful, and approachable. As leaders of the program, they are seen as humble, very hard working, assigning credit to EMBARC’s accomplishments to others, finding job leads, connecting students to important professionals, and generous with respect to time, check-ins, seen universally as trusted mentors. Anthony, a Latino student, noted that the PIs “*were always so warm and welcoming, and I never felt stupid or I never felt like I didn’t belong there*”.

#### 3.4.2. Realizing the Potential of Faculty-Student Relationships: Planning for a STEMM Career

Stories from professional panelists and faculty inspired students to imagine assuming a career that mirrored their own aspirations. For example, one student was inspired by a physician who was also a mother and a researcher; another student was impressed by a researcher who, combining disciplinary fields, freed the student to think about careers that integrate their interests. The task of becoming a STEMM professional seemed less “daunting” and more manageable once they had heard the actual stories of people like themselves, fueling a sense of confidence and self-efficacy. According to Melanie, a non-binary Asian/Asian American student, “*most of the professors that came to speak at the workshops, they really did make me feel inspired to pursue science and more confident in my ability*”.

Faculty-student relationships that were reframed appeared to have notable consequences for EMBARC students. Students described faculty increasing their interest (often described as eye-opening) in STEMM topics and careers by introducing them to specific topics (DNA extraction, hematology, genetics), professional development (CV and LinkedIn, self-representation), and by encouraging them to consider that a STEMM career was within their grasp. Betty, a Latina participant, stated, “*I saw people like me*. … *Representation is one of the things that drives people the most because it is like, ‘oh, someone like me did that. I can do it too’*”.

Faculty accessibility and trust was associated with students being less hesitant to contact other professionals, and students reported feeling more engaged on campus because they were more likely to communicate with others while on campus—and less likely to come to class and go home without meaningful interactions with others. Students described themselves as more confident and certain of their career pathway, empowered to overcome obstacles and draw on their community cultural wealth, peer and faculty networks to stay on course as a result of their interactions with faculty and other professionals.

Students described almost infectious energy from mentors within EMBARC—sharing passion, optimism, interest in social justice, and a calm and reassuring context that holistically considers student needs as they transfer to a four-year institution as a STEMM student. Having a community of knowledgeable, caring others emboldened students to believe more in themselves and in their future. Students further indicated they were motivated to explore new educational and career pathways because they saw themselves as more capable and as part of something bigger than themselves.

### 3.5. Navigating Cultural Mismatch

For most students, the summer program marked their first exposure to the concepts of cultural mismatch and community cultural wealth (CCW), offering a unique opportunity to reflect on how their backgrounds have shaped their STEMM journey. The concepts reflected students’ experiences navigating the tension between family expectations and academic demands and how they make sense of competing cultural worlds. Participation in the program also supported shifts in understanding, validation, and sense of agency. The findings below illustrate students’ experiences of recognition, reframing, and growth as they navigated familial and academic ecosystems.

#### 3.5.1. Living with Cultural Tension: Guilt, Pressure, and Limited Language

Before participating in the program, many students described experiencing cultural mismatch without having the language to fully articulate it. Students often internalized feelings of guilt, pressure, and isolation as personal struggles rather than contextual challenges shaped by family expectations and structural barriers. Betty, a Latina participant, reported, “*I feel like sometimes when I pursue my education, I’m acting selfishly*.” These tensions were frequently intensified by caregiving responsibilities and limited family familiarity with higher education. For example, Margaret, a female Asian/Asian American participant, reflected on the competing expectations of caregiving and academic success:

“I’m essentially my dad’s caretaker for the last three years of my life… [A]t the same time my parents … expect me to do that, but they also expect me to succeed and do very well in school…. [T]hey haven’t been to school, so they don’t actually understand the difficulty of it.”

Similarly, Christopher, a Latino student, described the pressure to prioritize work over education and the lack of understanding within his family context:

“Because the cultural mismatch is my family…. Other than that, it was just like, you got to work, you got to do this. What’s the degree going to give you this? … It’s a completely different world than what we’re told.”

These examples underscore a student’s internalization of societal and familial pressures without recognizing the structural and cultural systems shaping their experiences. Liberation Psychology frames this as a crucial moment for cultivating awareness: students must first recognize these systemic and intergenerational constraints before transformative work can occur.

#### 3.5.2. Naming the Experience: Validation and Shared Understanding

Through participation in the program, students encountered structured opportunities to reflect on their experiences and hear them named in ways that resonated deeply. Program modules, discussions, and storytelling activities created space for students to recognize that their struggles were shared and rooted in broader cultural and social contexts. Barbara, a white female participant, described a moment of awakening when the concept of cultural mismatch was introduced:

“One of the first modules … was cool, like cultural mismatch, because it kind of hit the nail on the wall for me for the longest time…. I was like, oh, so that’s what the hell was happening. It kind of opened my eyes and I feel enlightened.”

For many students, this naming process functioned as a critical consciousness-raising tool, allowing them to reframe long-standing stressors as structural rather than personal and reducing feelings of isolation and self-blame. Group-based activities further reinforced this sense of shared understanding. Gene, a female Middle Eastern or North African participant, reflected on how storytelling exercises during the Ancestral Strengths workshop created connection and emotional safety within the cohort: “*We went around* … *talking about our past*. … *I feel like that really brought us together as a group*”.

Through these activities, students began to understand that their experiences of guilt, stress, or dissonance were not isolated failings, but products of broader social, cultural, and historical ecosystems. Being able to situate their challenges within collective histories and cultural narratives has reportedly fostered validation and mutual support.

#### 3.5.3. Moving Forward with Clarity and Agency

As students continued in the program, many described shifts in how they understood their roles within both family and academic spaces. Gaining language and practical strategies for cultural mismatch allowed students to reframe their experiences, feel more grounded in their educational journeys, and engage with college life more intentionally. Referring to workshops that engage with concepts of cultural mismatch and CCW, Christopher, a Latino participant, revealed how this newfound awareness translated into a sense of agency: “*Being educated on those workshops* … *they help me connect with this whole college experience and gaining this world familiarity* [sic]”.

By being introduced to the concept of cultural mismatch and receiving tools to navigate it, students reported feeling better able to navigate family expectations while maintaining their academic goals. These experiences were described by participants as enabling them to reclaim their narratives, increase confidence, reduce self-blame, and foster the development of supportive peer networks, which they associated with approaching their education with greater clarity and purpose.

### 3.6. Benefits to Students of Living on the University Campus

Across five focus groups, students described living on-campus as core program benefit, providing an immersive “practice experience” for the four-year college life. Dorming helped students understand the day-to-day lived realities of being a residential student, increased confidence, and generated a sense of independence.

#### 3.6.1. Understanding the College Experience Through Immersion

Students frequently framed dorming as the difference between hearing about college and actually living it. The residential experience offered an authentic lived experience regarding what it is like to be a college student. As Emily, a female Middle Eastern or North African participant, described, “*You get to know how it’s going to work when you’re actually here. That’s the key part* … *learning how to coordinate everything. Being on the campus*”.

While living in the dorms, students were able to practice sharing space with peers, navigating campus routines, and managing day-to-day responsibilities in a university environment.

Dorming also made the transfer pathway feel more concrete. Students described gaining practical familiarity with campus life. They experienced how to move through a university’s unfamiliar spaces, coordinate schedules, and anticipate the demands of “adulting” in an academic setting. Sandy, a Latina student, stated,

“I think it definitely helps you feel more of a sense of maturity, like you’re doing all of these things on your own and preparing this for adulthood and for when we transfer … and I think that can be a little scary.”

Importantly, students characterized dorming as a low-stakes and immersive way to experience residential college life before making longer-term decisions about transfer.

#### 3.6.2. Confidence, Independence, and College-Student Identity

Living on campus supported students’ confidence by giving them lived evidence that they can navigate college life. This type of experience is especially important for transfer students who are not always familiar with the requisites for success at a four-year university. Students in the program described dorming as creating a meaningful shift in self-efficacy, giving them a newfound opportunity to manage daily routines, experience independence, and handle new responsibilities. As Betty, a Latina student, described, EMBARC “*gave me an opportunity to experience new things that I never thought I would experience*, … *I felt really homesick when I came here and I didn’t appreciate some aspects of living here, but I did like it here*”.

Some students, like Linda, a Latina participant, described a broader “eye-opening” transformation in mindset, from uncertainty to readiness, consistent with developing college-student identity, and an increased sense of confidence, and independence to pursue opportunities:

“I just have learned so much about myself … I’m learning that I’m capable of doing things that I am scared of and not just now, but in the future … I feel it’s just eye opening for me personally.”

Overall, students emphasized that the residential context made them feel more confident in navigating the uncertainties in becoming a successful university student and generated a transformation in identity to envision themselves as a four-year university student.

#### 3.6.3. Reduced Uncertainties and Supporting Basic Needs: More Time and Energy for Learning and Engagement

Beyond the social and identity benefits, students also described dorming as reducing daily uncertainties that can undermine academic success. Being on campus helped students stay focused instead of being pulled back into their outside stressors and responsibilities. Andrew, a Latino participant, explained that leaving and returning to “the real world” would have disrupted his immersion in the experience:

“[I]f we were off campus … that’s going to re-engage us back into the open world where I have all these bills, or I have to deal with this or … give this person my attention. …I want to be … focused on what I have to do here and being able to fully submerge in the experience.”

By removing commuting pressures and making food and basic needs more predictable, living on campus reduced uncertainty and increased punctuality, focus, and the capacity to make peer connections. These benefits were especially pronounced for students who imagined themselves transferring to a university setting. As Margaret, a female Asian/Asian American student, described:

“I think this program is just really great to show people the taste of what it’s like to be in a dorm, but also how to actually navigate through higher education.”

Like Andrew and Margaret, students connected their dorming experience to routine-building and reducing uncertainties, factors that can be especially important for students in learning to balance multiple responsibilities.

### 3.7. Validation and Commitment to Work in STEMM

Despite aspirations, underrepresented students are known to leave the STEMM pathway more frequently than their peers due to factors such as transfer shock, lack of representation, and limited resources, as discussed in the introduction. The goal of EMBARC, therefore, is to address these gaps by providing support at all levels—including peer and faculty mentorship, stipends, transfer and research resources, and attention to cultural needs—to strengthen persistence, degree completion, and retention in STEMM pathways.

#### 3.7.1. Increased Drive to Continue in STEMM

In the focus groups, several students reflected on how the program reassured them that they were on the right path in STEMM. For example, Cynthia, a Latina student, reflected, “*I saw a lot of examples and I met a lot of people who made me understand that I’m doing the right thing*…*. It gave me a sense of security*.” Other students described an increased drive to continue in STEMM and a strengthened passion for research through various program components, including lab tours, faculty presentations, skills-building activities, validation from mentors, and exposure to representation. Repeatedly, students mentioned that they were deeply inspired by the faculty presentations, where faculty shared their personal journeys and research. Referring to a professor who engages in research and has four kids, Margaret, a female Asian/Asian American student, said,

“[She] literally does everything I feel like that you could have ever possibly do. There’s nothing that she doesn’t do and that was just cool to see. And yeah, I just felt very motivated…. [I]n 10 years … I actually can see me getting there.”

Through role models, students came to believe that they, too, could pursue science, attend graduate school, and envision themselves as accomplished scientists.

#### 3.7.2. Humanizing STEMM and Grounding Success in Relational Frameworks

“I would say even though it’s kind of STEMM oriented, it also includes the cultural aspect because it’s not like it’s just all cold STEMM research or no feelings, but it’s like people. They come from different backgrounds and you should really honor that in their experiences. And I feel like we are able to see where our background plays into our lives now and how that affects our values and our future trajectory. I like that part.”

(Melanie, Non-binary, Asian/Asian American)

The incorporation of ancestral strengths, cultural mismatch, and community cultural wealth makes EMBARC stand out among similar programs that aim to help community college students pursue and persist in STEMM, grounding motivation in relational and ethical frameworks rather than solely in individual achievement. As Melanie has rightly observed, one’s cultural backgrounds shape their everyday life and moral values and are integral to their education and career pursuits. Additionally, aspects of one’s cultural wealth, such as bilingualism and resilience, are all recognized as valuable STEMM competencies in EMBARC. As such, any discussions about STEMM careers that do not recognize one’s cultural wealth or ancestral legacies fail to guide students in drawing on their internal wisdom and strengths, which may be their greatest sources of resilience on a challenging path. Humanizing STEMM not only portrays a more holistic picture of the field but also demystifies and challenges the preconceived notion of STEMM as a “cold” career, enabling students to more readily see themselves in the role models and envision themselves on this career path.

Note that, although there is no dedicated section on CCW in our findings, the concept underpins our results, as the cultural components serve as foundational elements throughout the program. Students engage with CCW capitals in multiple ways. For example, they reflected on their familial capital while exploring their ancestral roots and strengths, exercised their social capital through peer interactions and mentoring relationships, and developed navigational skills as they manage on-campus living and the transfer pathway. Similarly, while Section 3.5 highlights students gaining strategies for navigating cultural mismatch, its practical application extends beyond individual instances. It encompasses the ability to navigate the broader academic context (e.g., acquiring knowledge of transfer processes and relevant resources, building connections with faculty, and gaining confidence in their belonging in STEMM despite deficit messages). The program’s holistic approach can thus be understood as a comprehensive response to cultural mismatch.

Although EMBARC was overwhelmingly praised for its utility—with students exclaiming that the resources provided exceeded their expectations—the program was not without limitations. For example, some students indicated that the program’s scheduling could be better structured, as certain sessions felt rushed and did not allow sufficient time to complete activities such as developing an Individual Development Plan, setting SMART goals, building CVs/resumes, and conducting data analysis. Additionally, although students found the faculty presentations inspiring, some suggested that having fewer presenters per session would be beneficial, allowing each presenter more time to thoroughly present their material. The larger size of the third cohort further limited students’ ability to form the close relationships experienced by earlier cohorts; consequently, students from this cohort recommended implementing intentional and meaningful community-building activities, rather than leaving them as optional. Lastly, readers should be reminded of the program’s temporal scope: because data were collected immediately after program completion, the findings reflect students’ short-term perceptions. Longer-term tracking will be necessary to evaluate the program’s sustained impact.

## 4. Discussion

The goal of this study was to examine how EMBARC impacts students’ desire to pursue a STEMM career, their sense of belonging and ability to complete STEMM preparation, and their identity as a scientist or practitioner. By directly addressing students’ cultural strengths and passions for science, including social justice research, EMBARC invited students to be their authentic selves, to see structural barriers as surmountable through community and hard work, and to develop the skills to envision and plan for their own pathway with realistic knowledge, confidence, and a belief that they have much to contribute to science.

### 4.1. Theoretical Implications: Awareness of Historical and Cultural Factors Shapes Student STEMM Motivation

From a Liberation Psychology lens, recognizing systemic barriers and leveraging cultural roots are critical to culturally responsive mentoring, as this framework centers historical, collective, and cultural sources of knowledge and resilience (Castellanos et al., 2023). At a systemic level, institutional investments in hands-on experiences and community-building opportunities (e.g., campus housing or summer intensive programs) can reduce transfer stigma and cultural mismatch. Through these supports, EMBARC participants reported increased academic and scientific confidence (see also Guan et al., 2024). They also experienced strengthened identities, including a deeper connection to and validation of their cultural and ancestral backgrounds.

By creating systemic supports that facilitate the integration of students’ full identities into their STEMM journeys, we observed an evolution among EMBARC participants—from limited awareness of academic norms and systemic barriers to a deeper understanding of how to navigate them, and ultimately to growing agency in shaping their educational paths. Together, these experiences transformed students’ perceptions of academic systems from mysterious and intimidating into navigable and negotiable spaces. As such, EMBARC serves as a programmatic model for honoring and leveraging students’ full identities, including their ancestral strengths, which function as a foundational—yet often underrecognized—source of motivation.

### 4.2. Methodological Implications: Multi-Tiered and Humanizing Mentorship Reduces Fears and Increases Scientific Self-Efficacy

From “seeing” and humanizing the faculty members to “being seen” by them, our findings show that mentoring was a key source of student empowerment and transformation. Students experienced mentors as steady relational anchors who modeled both professional competence and emotional openness—this was especially powerful in seeing mentors’ full identities beyond their titles. The impact of mentoring extended to peer interactions, where students saw themselves as part of a group and learned from one another’s journeys. Established trust, reduced fears, and an increased sense of belonging were associated with science self-efficacy and identity among students. Students who became peer navigators then passed on this modeling to others, fostering a stronger sense of personal agency in both themselves and those they guided. In this way, students grow professionally and socioemotionally—not only through interactions with faculty and peers but also through helping others navigate their journeys as peer navigators. The program, therefore, exemplifies a “multigenerational mentoring model” that leverages the combined strengths of faculty, peer navigators, and student participants at large (Castellanos et al., 2023). Studies have shown that well-established peer navigating programs are an effective strategy for increasing retention rates, students’ sense of well-being and belonging, and, to a lesser extent, academic performance in higher education (Lane, 2020; Le et al., 2024; Lorenzetti et al., 2019; Sanchez et al., 2006; Tomlinson & Cameron, 2025; Yomtov et al., 2017). Our findings add to the growing evidence that community college students in general and peer navigators in particular gain academic, interpersonal, and professional benefits from these experiences—at times, life-transforming ones (Abeywardana et al., 2020; Dixon et al., 2023; Ghosh & Reio, 2013; Morales et al., 2020).

### 4.3. Practical Implications: Training Mentors to Leverage Cultural Capital to Support Student STEMM Success

Rising concern about America’s ability to maintain its competitive position in the global economy has prompted calls for the U.S. higher education system to produce more graduates with training and expertise in STEMM fields (Olson & Riordan, 2012). To attain this goal, policymakers recommend reducing STEMM attrition in college, arguing that retaining more students in the fields in college is one way to expand the pool of STEMM professionals that the nation needs to advance economically and be globally competitive. Based on our research findings, this study argues that mentorship programs that affirm ancestral knowledge and cultural heritage through “multidimensional mentorship models” can transform STEMM culture. Such programs can shift the focus from one that emphasizes “publish or perish,” disadvantages marginalized groups, and stifles innovation, to one that emphasizes “publish and flourish,” values marginalized voices and experiences, and fosters STEMM innovation (Davies et al., 2021). Our results further indicate that interactions emphasizing representation, relational trust, and emotional affirmation are central to identity development and self-transformation. These findings underscore the importance of training mentors to explicitly reflect on and leverage their cultural capital, such as knowledge, skills, and lived experiences, when guiding others. As highlighted in the study, discussions about STEMM careers that fail to recognize students’ cultural wealth or ancestral legacies risk overlooking their internal wisdom and strengths, which may be their greatest sources of resilience on a challenging path.

### 4.4. Limitations & Future Directions

While EMBARC participants have generally reported positive experiences with the program, EMBARC served students in Southern California primarily at HSI/MSI institutions; therefore, scaling programs like it will require examining how the intervention can be effectively implemented in other regions with different sociodemographics, student populations, educational policies and cultures (e.g., the Southern U.S.). That said, we believe that for any programs serving student populations underrepresented in STEMM fields, the elements discussed in this article—particularly learning about multiple pathways through faculty members’ academic journeys, feeling a sense of belonging through bonding with peers, and gaining the language to describe home–school mismatch experiences—are transferable to other settings.

Another limitation concerns funding support. EMBARC students received a stipend to attend the two-week summer program, which helped meet their basic needs, as noted by some students in the focus groups. Without a stipend, students may not have considered the opportunity, as economic pressures might have forced them to take on a job. The lack of funding may create an institutional constraint that limits others’ ability to replicate the program.

A further limitation involves the temporal scope of the program. Because data were collected immediately after program completion, the findings from this study reflect students’ short-term perceptions and do not confirm the program’s sustained impact.

Increasing federal funding for the development of community college programs to populate STEMM niches with diverse scientists while supporting their local control has great potential to yield a high return-on-investment because of the untapped talent and diversity of community college students. Existing programs that publish their findings support a broader community of researchers and educators who are transforming student training programs to better meet the needs of our current student populations. At the time of writing, EMBARC is developing online modules that will ultimately be made available to community college professors and administrators, as well as to those interested in fostering a transfer-receptive culture at four-year and other higher education institutions. Building a broad database of training program successes contributes to the overall well-being of our students and, indeed, our country, in preparing for the future while honoring the talent in our country that is often overlooked.

## Figures and Tables

**Table 1.** Demographic characteristics of participants.

Characteristic	Frequency	Percentage
College Generation		
First-Generation College Student	31	72
Continuing-Generation College Student	12	28
Gender Identity		
Female	31	72
Male	11	26
Non-binary	1	2
Race/Ethnicity		
African American/Black	2	5
Asian American	10	23
Latina/o/x	24	56
Middle Eastern or North African	5	12
Multi-Race		
Other		
White	2	5
Field of Study		
Engineering & Computer Science	8	19
Health & Human Development	4	9
Science & Math	12	28
Social & Behavioral Sciences	19	44
Total	43	100

**Table 2.** Main themes and subthemes identified.

Main Themes	Subthemes
Guidance, Mentorship, and Navigation	Dissonance without Structural Frameworks
	Guidance as Meaning Making: From Passive Receipt to Active Co-Construction
Acquisition of Knowledge, Hard Skills, and the Hidden Curriculum	Anxiety and Self-Doubt Stemming from Limited Access
	Expanding Academic Capacity through Exposure to Diverse Tools and Skills
Peer Bonding as a Foundation for Belonging	Vulnerability Fosters Emotionally Powerful Connections
	From Bonding to Belonging Through Trust and Safety
	From Belonging to Embracing Challenges in Team Science
Mindset Shifts: Preparing for the Future and Faculty-Student Relationships	Unlocking the Potential of Faculty-Student Relationships: Openings for the Future
	Realizing the Potential of Faculty-Student Relationships: Planning for a STEMM Career
Navigating Cultural Mismatch	Living with Cultural Tension: Guilt, Pressure, and Limited Language
	Naming the Experience: Validation and Shared Understanding
	Moving Forward with Clarity and Agency
Benefits to Students of Living on the University Campus	Understanding the College Experience Through Immersion
	Confidence, Independence, and College-Student Identity
	Reduced Uncertainties and Supporting Basic Needs: More Time and Energy for Learning and Engagement
Validation and Commitment to Work in STEMM	Increased Drive to Continue in STEMM
	Humanizing STEMM and Grounding Success in Relational Frameworks

## Data Availability

The data supporting this study are not publicly available to protect the confidentiality and anonymity of the participants, as guaranteed through the informed consent process.

## References

[R1] Abeywardana SU, Velasco S, Hall N, Dillon J, & Chun CA (2020). Near-peer mentoring in an undergraduate research training program at a large Master’s comprehensive institution: The case of CSULB BUILD. UI Journal, 11(1), 12477. 35558987 PMC9094756

[R2] American Association of Community Colleges. (n.d.). DataPoints: Recognizing student veterans’ challenges. Available online: https://www.ccdaily.com/2021/11/datapoints-recognizing-student-veterans-challenges/ (accessed on 26 January 2026).

[R3] American Association of Community Colleges. (2025). Fast facts 2025. Available online: https://www.ccdaily.com/2025/02/aacc-fast-facts-2025/ (accessed on 26 January 2026).

[R4] Anzaldúa G (1987). Borderlands: The new mestiza = La frontera (1st ed.). Spinsters/Aunt Lute.

[R5] Beals R, Zimny S, Lyons F, & Bobbitt O (2021). Activating social capital: How peer and socio-emotional mentoring facilitate resilience and success for community college students. Frontiers in Educatio, 6, 667869.

[R6] Bell A, Chetty R, Jaravel X, Petkova N, & Van Reenen J (2019). Who becomes an inventor in America? The importance of exposure to innovation. The Quarterly Journal of Economics, 134(2), 647–713.

[R7] Bowling B, Doyle M, Taylor J, & Antes A (2015). Professionalizing the role of peer leaders in STEM. Journal of STEM Education,16(2), 30.

[R8] Cantor JC, Miles EL, Baker LC, & Barker DC (1996). Physician service to the underserved: Implications for affirmative action in medical education. Inquiry, 33(2), 167–180. 8675280

[R9] Castellanos J, White JL, & Franco V (2023). Riding the academic freedom train: A culturally responsive, multigenerational mentoring model. Routledge.

[R10] Cejda BD (2019). An overview of undergraduate research in community colleges. In Cejda BD, & Hensel N (Eds.), Undergraduate research at community colleges (pp. 1–7). Council on Undergraduate Research.

[R11] Charmaz K (2014). Constructing grounded theory (2nd ed.). Sage.

[R12] Chen X (2005). First-generation students in postsecondary education: A look at their college transcripts. Postsecondary education descriptive analysis report (NCES 2005–171). U.S. Department of Education, National Center for Education Statistics. Available online: https://nces.ed.gov/pubs2005/2005171.pdf (accessed on 26 January 2026).

[R13] Chen X, & Soldner M (2013). STEM attrition: College students’ paths into and out of stem fields. Statistical analysis report (NCES 2014–001). U.S. Department of Education, National Center for Education Statistics. Available online: https://nces.ed.gov/pubs2014/2014001rev.pdf (accessed on 26 January 2026).

[R14] Cohen JJ, Gabriel BA, & Terrell C (2002). The case for diversity in the health care workforce. Health Affairs, 21(5), 90–102. 12224912 10.1377/hlthaff.21.5.90

[R15] Collins PH (1986). Learning from the outsider within: The sociological significance of black feminist thought. Social Problems, 33(6), S14–S32.

[R16] Community College Research Center. (n.d.). Community college FAQs. Teachers College, Columbia University. Available online: https://ccrc.tc.columbia.edu/community-college-faqs.html (accessed on 26 January 2026).

[R17] Davies SW, Putnam HM, Ainsworth T, Baum JK, Bove CB, Crosby SC, Côté IM, Duplouy A, Fulweiler RW, Griffin AJ, Hanley TC, Hill T, Humanes A, Mangubhai S, Metaxas A, Parker LM, Rivera HE, Silbiger NJ, Smith NS, … Bates AE (2021). Promoting inclusive metrics of success and impact to dismantle a discriminatory reward system in science. PLoS Biology, 19(6), e3001282. 34129646 10.1371/journal.pbio.3001282 PMC8205123

[R18] Davis G (2005). Doctors without orders. American Scientist, 93(3), S1–S13.

[R19] Dixon BT, Agboola O, Hauck A, Argento M, Miller C, & Vaughan AL (2023). Peer mentoring: Benefits to first-time college students and their peer mentors. Journal of Higher Education Theory and Practice, 23(2), 202–217.

[R20] Fry R, Kennedy B, & Funk C (2021). STEM jobs see uneven progress in increasing gender, racial and ethnic diversity. Pew Research Center.

[R21] Garde-Hansen J, & Calvert B (2007). Developing a research culture in the undergraduate curriculum. Active Learning in Higher Education,8(2), 105–116.

[R22] Ghosh R, & Reio TG Jr. (2013). Career benefits associated with mentoring for mentors: A meta-analysis. Journal of Vocational Behavior, 83(1), 106–116.

[R23] Guan S-SA, Ashcroft J, Horowitz B, Ie E, Vasquez-Salgado Y, & Saetermoe C (2024). Sociocultural and contextual determinants of science career goal at a community college and baccalaureate-granting institution. International Journal for Educational and Vocational Guidance, 24(1), 59–75. 38725969 10.1007/s10775-022-09547-x PMC11081598

[R24] Guan S-SA, & Vasquez-Salgado Y (2023). A cultural mismatch intervention to increase science self-efficacy among STEM college students. UI Journal, 14(2). PMC10836422 38312986

[R25] Guan S-SA, Vasquez-Salgado Y, Saetermoe CL, Lin JCP, Bui AKA, Villaseñor V, Boyns D, Ashcroft J, Haynes-Mendez K, & Swales-Jefferson L (2025). Design, development, and impact of educational modules to broaden academic research cultures (EMBARC) for STEMM career development: A pilot project. Journal of Advanced Technological Education, 4(1), 10–5281. 10.5281/zenodo.15587802 PMC12345232 40810111

[R26] Hall JN, Avent CM, Boyce AS, & Acheampong KO (2023). Culturally responsive evaluation teaching and learning in higher education: A higher calling. New Directions for Evaluation, 2023(180), 85–92.

[R27] Hewlett J (2019). The search for synergy: Undergraduate research at the community college. In Cejda BD, & Hensel N (Eds.),Undergraduate research at community colleges (pp. 8–18). Council on Undergraduate Research.

[R28] Hobin JA, Clifford PS, Dunn BM, Rich S, & Justement LB (2014). Putting PhDs to work: Career planning for today’s scientist. CBE Life Sciences Education, 13(1), 49–53. 24591503 10.1187/cbe-13-04-0085 PMC3940462

[R29] Hooks B (1994). Teaching to transgress: Education as the practice of freedom. Routledge.

[R30] Hutchison L, & McAlister-Shields L (2020). Culturally responsive teaching: Its application in higher education environments. Education Sciences, 10(5), 124.

[R31] Institute of Medicine. (2004). In the nation’s compelling interest: Ensuring diversity in the health-care workforce. The National Academies Press. 25009857

[R32] Judge J, Lannon HJL, Stofer KA, Matyas CJ, Lanman B, Leissing JJ, Rivera N, Norton H, & Hom B (2022). Integrated academic, research, and professional experiences for 2-year college students lowered barriers in STEM engagement: A case study in geosciences. Journal of STEM Outreach, 5(1), 1–15.

[R33] Lane SR (2020). Addressing the stressful first year in college: Could peer mentoring be a critical strategy? Journal of College Student Retention: Research, Theory & Practice, 22(3), 481–496.

[R34] LaVeist TA, & Nuru-Jeter A (2002). Is Doctor-patient race concordance associated with greater satisfaction with care? Journal of Health and Social Behavior, 43(3), 296–306. 12467254

[R35] Le H-G, Sok S, & Heng K (2024). The benefits of peer mentoring in higher education: Findings from a systematic review. Journal of Learning Development in Higher Education, (31).

[R36] Lin JCP, Moon S, Guan S-SA, Kwan PP, Flores GE, & Chavira G (2025). Factors influencing science career intention: The power of counterspace. Innovative Higher Education, 50(2), 641–663. 40255937 10.1007/s10755-024-09752-2 PMC12003595

[R37] Lorenzetti DL, Shipton L, Nowell L, Jacobsen M, Lorenzetti L, Clancy T, & Paolucci EO (2019). A systematic review of graduate student peer mentorship in academia. Mentoring & Tutoring: Partnership in Learning, 27(5), 549–576.

[R38] Macapugay K, & Nakamura B (2024). The student empowerment through narrative, storytelling, engagement, and identity framework for student and community empowerment: A culturally affirming pedagogy. Genealogy, 8(3), 94.

[R39] Martín-Baró I, Aron A, & Corne S (1994). Writings for a liberation psychology. Harvard University Press.

[R40] Meza EA (2019). Underserved community college students and the complexity of STEM transfer (Data Note 1, STEM Series). Community College Research Initiatives.

[R41] Morales DX, Wagler AE, & Monarrez A (2020). BUILD peer mentor training model: Developing a structured peer-to-peer mentoring training for biomedical undergraduate researchers. UI Journal, 11(1). Available online: https://www.understandinginterventionsjournal.org/article/12480-build-peer-mentor-training-model-developing-a-structured-peer-to-peer-mentoring-training-for-biomedical-undergraduate-researchers (accessed on 26 January 2026). PMC7467556 32885224

[R42] National Academies of Sciences, Engineering, and Medicine (NASEM). (2016). Barriers and opportunities for 2-year and 4-year stem degrees:Systemic change to support students’ diverse pathways. The National Academies Press. 27336114

[R43] National Academies of Sciences, Engineering, and Medicine (NASEM). (2017). Undergraduate research experiences for STEM students: Successes, challenges, and opportunities. The National Academies Press.

[R44] National Science Board, National Science Foundation. (2024). The STEM labor force: Scientists, engineers, and technical workers (Science and Engineering Indicators 2024. NSB-2024–5). National Science Board, National Science Foundation. Available online: https://ncses.nsf.gov/pubs/nsb20245/ (accessed on 26 January 2026).

[R45] Olson S, & Riordan DG (2012). Engage to excel: Producing one million additional college graduates with degrees in science, technology, engineering, and mathematics (Report to the President). Executive Office of the President.

[R46] Richardson L, & St. Pierre EA (2005). Writing: A method of inquiry. In Denzin NK, & Lincoln YS (Eds.), The Sage handbook of qualitative research (3rd ed., pp. 959–978). Sage.

[R47] Rockinson-Szapkiw A, Wendt JL, & Stephen JS (2021). The efficacy of a blended peer mentoring experience for racial and ethnic minority women in stem pilot study: Academic, professional, and psychosocial outcomes for mentors and mentees. Journal for STEM Education Research, 4(2), 173–193.

[R48] Saetermoe CL, Vargas JH, Paez JM, & Garrow W (2025). Liberatory race-conscious mentorship practices: Collaborating for change. Action in Teacher Education, 47(3), 208–227. 40822868 10.1080/01626620.2024.2430767 PMC12356013

[R49] Sanchez RJ, Bauer TN, & Paronto ME (2006). Peer-mentoring freshmen: Implications for satisfaction, commitment, and retention to graduation. Academy of Management Learning & Education, 5(1), 25–37.

[R50] Snyder J, & Cudney EA (2017). Retention models for stem majors and alignment to community colleges: A review of the literature. Journal of STEM Education, 18(3), 48–57.

[R51] Spaulding DT, Kennedy JA, Rozsavolgyi A, & Colón W (2020). Outcomes for peer-based mentors in a university-wide stem persistence program: A three-year analysis. Journal of College Science Teaching, 49(4), 30–36.

[R52] Stephens NM, Fryberg SA, Markus HR, Johnson CS, & Covarrubias R (2012). Unseen disadvantage: How American universities’ focus on independence undermines the academic performance of first-generation college students. Journal of Personality and Social Psychology, 102(6), 1178–1197. 22390227 10.1037/a0027143

[R53] Strauss AL, & Corbin JM (1998). Basics of qualitative research: Techniques and procedures for developing grounded theory (2nd ed.). Sage.

[R54] Tomlinson AN, & Cameron NG (2025). Association of peer mentoring on nursing student retention: A systematic literature review. Journal of Nursing Education, 64(5), 294–298. 40332994 10.3928/01484834-20250108-08

[R55] Vargas JH, & Saetermoe CL (2024). The antiracist educator’s journey and the psychology of critical consciousness development: A new roadmap. Educational Psychologist, 59(1), 20–41.

[R56] Vasquez-Salgado Y, Greenfield PM, & Angie Guan S-S (2021). Home-school cultural value mismatch: Antecedents and consequences in a multi-ethnic sample transitioning to college. Frontiers in Psychology, 12, 618479. 34552520 10.3389/fpsyg.2021.618479 PMC8450350

[R57] Vasquez-Salgado Y, Greenfield PM, Angie Guan S-S, Gonzalez L, & Tarlow DA (2022). Peer-peer cultural value mismatch in the dormitory during the transition to college: Antecedents and correlates. Journal of Intercultural Communication & Interactions Research, 2(1), 37–74. 10.3726/jicir.2022.1.0004 PMC10392955 37529117

[R58] Vasquez-Salgado Y, Halim G, Morales AE, Galvan K, Seeman T, & Cole SW (2025). Cultural mismatch and accelerated epigenetic age during the transition to college. Brain, Behavior, & Immunity—Health, 46, 100989. 10.1016/j.bbih.2025.100989 PMC12155781 40502527

[R59] Warren JM, Goins CL, Locklear LA, Unger DL, Locklear TM, Neal G, Nickolson C, & Robinson GG (2020). Culturally responsive perceptions and practices of instructors at a minority-serving institution: A mixed methods study. Journal of Effective Teaching in Higher Education, 3(2), 76–91.

[R60] Yomtov D, Plunkett SW, Efrat R, & Marin AG (2017). Can peer mentors improve first-year experiences of university students? Journal of College Student Retention: Research, Theory & Practice, 19(1), 25–44.

[R61] Yosso TJ (2005). Whose culture has capital? A critical race theory discussion of community cultural wealth. Race, Ethnicity and Education, 8(1), 69–91.

[R62] Zaniewski AM, & Reinholz D (2016). Increasing STEM success: A near-peer mentoring program in the physical sciences. International Journal of STEM Education, 3(1), 14.

